# 2530 Relationship between preexisting pain and completion of a community-based wellness program

**DOI:** 10.1017/cts.2018.261

**Published:** 2018-11-21

**Authors:** Chantay Young, Sifang Zhao, Tash Weddle, Sarah Jones, Digna Velez-Edwards, Katherine Hartmann

**Affiliations:** 1 New Beginnings; 2 Vanderbilt University Medical Center

## Abstract

OBJECTIVES/SPECIFIC AIMS: New Beginnings is a 12-week community-based behavioral intervention for improving health, strength, and wellness through a holistic approach to coaching that supports lifestyle change. The program serves predominantly low-income, minority women. Given the substantial focus on exercise, including resistance training, we aimed to test whether pain at baseline is associated with program completion in a prospective cohort. METHODS/STUDY POPULATION: At the entry of the New Beginnings program, women completed a survey that included a body map of sites at which they experienced pain for most days in the prior week. Using logistic regression, we independently tested the association between presence of pain, the total number of pain sites, and grouped location of pain with program completion, assessing the following a priori candidate confounders: age, race/ethnicity, body mass index, and income. We also tested for interaction of pain and age in influencing completion. RESULTS/ANTICIPATED RESULTS: Seventy-five percent of participants, 185 of 247, completed the program. They had an average age of 44.2±11.7 years, weight of 244.5±115.4 pounds, and BMI of 41.3±18.2. Fifty-seven percent were African American and 3% were Hispanic. The majority reported preexisting pain (83%), with an average of 3.4±2.7 pain sites. Completers and non-completers did not differ by the total number of pain sites (*p*=0.2). Having preexisting pain compared to no pain [odds ratio (OR)=1.3; 95% confidence interval (CI): 0.5–3.4] and to the number of pain sites (OR=1.0; 95% CI: 0.9–1.1) did not influence program completion after adjusting for the sole confounder, which was age. Likewise, we observed no association between limb/joint pain (OR=1.1; 95% CI: 0.6–2.1) or back pain (OR=0.9; 95% CI: 0.5–1.6) with program completion. The association of pain with completion was not modified by age. DISCUSSION/SIGNIFICANCE OF IMPACT: While pain is believed to be a barrier to improving fitness, preexisting pain may not be a strong predictor of completing a holistic lifestyle intervention with a substantial exercise component. Rather, women’s commitment to making a healthy lifestyle change may result in program completion irrespective of preexisting pain. Addressing and accommodating pain-related modifications to exercise interventions promise to be more effective than excluding those with pain from participation.

